# Widespread Prevalence of Plasmid-Mediated Colistin Resistance Gene *mcr-1* in Escherichia coli from Père David's Deer in China

**DOI:** 10.1128/mSphere.01221-20

**Published:** 2020-12-23

**Authors:** Xiaoyu Lu, Xia Xiao, Yuan Liu, Siyang Huang, Ruichao Li, Zhiqiang Wang

**Affiliations:** a Jiangsu Co-Innovation Center for Prevention and Control of Important Animal Infectious Diseases and Zoonoses, College of Veterinary Medicine, Yangzhou University, Yangzhou, Jiangsu, People’s Republic of China; b Institute of Comparative Medicine, Yangzhou University, Yangzhou, Jiangsu, People’s Republic of China; c Joint International Research Laboratory of Agriculture and Agri-Product Safety, Ministry of Education of China, Yangzhou University, Yangzhou, Jiangsu, People’s Republic of China; Antimicrobial Development Specialists, LLC

**Keywords:** *Escherichia coli*, IncI2, Père David's deer, *bla*_CTX-M-132_, colistin resistance, *mcr-1*, nanopore sequencing

## Abstract

Père David’s deer (Elaphurus davidianus or milu) is an endangered species, and the prevalence of antimicrobial resistance (AMR) such as *mcr-1*-positive strains among them has been unknown. In this study, we aimed to investigate the genomic characterizations of *mcr-1*-positive strains and provide insight into the dissemination of AMR in nature reserve settings. Sixty-seven *mcr-1*-positive Escherichia coli isolates from 97 fecal samples were identified by PCR and found resistant to colistin. The prevalence of β-lactam resistance was very high, and there were 64 *mcr-1*-positive isolates containing β-lactamase genes. Transconjugants of 66 *mcr-1*-positive isolates were acquired through conjugation experiments. PCR-based replicon typing (PBRT) showed that 44 strains harbored IncI2 *mcr-1*-bearing plasmids, eight strains harbored IncX4 *mcr-1*-carrying plasmids, and 14 strains harbored IncHI2 *mcr-1*-positive plasmids. Notably, *mcr-1* was located in the chromosome of LD27-1. Clonal dissemination and horizontal dissemination of *mcr-1* by plasmids coexist. We first report the prevalence of plasmid-mediated *mcr-1* in E. coli from Père David’s deer in China. *mcr-1*-bearing IncI2 plasmid was the most frequent plasmid type, and the first IncI2 plasmid harboring both *bla*_CTX-M-132_ and *mcr-1* is characterized here. Our results support the implication of Père David’s deer as a potential reservoir for MCR-1-producing E. coli.

**IMPORTANCE** The *mcr-1* gene is widely reported around the world and has been identified on various plasmids with different replicon types. Resistance to the last-line antibiotic colistin mediated by *mcr-1* still represents a threat to global public health. Père David’s deer is a highly endangered species originating in China, and many deer are currently being raised in captivity for gradual reintroduction to the wild. If this species carries AMR bacteria, it will pose a potential threat to the environment. Therefore, research on the dissemination of *mcr-1*-positive E. coli from Père David’s deer is of great significance. This is the first study to investigate the microbiological and genomic surveillance of MCR-1-producing bacteria colonized among Père David’s deer in China, and we uncovered a high prevalence of MCR-1-producing E. coli. The importance of constant surveillance for AMR bacteria in nature reserve settings is emphasized.

## Abstract (precis)

The *mcr-1* gene is widely reported around the world and has been identified on various plasmids with different replicon types. Resistance to the last-line antibiotic colistin mediated by *mcr-1* still represents a threat to global public health.

## OBSERVATION

Plasmid-mediated mobilized colistin resistance (*mcr*) genes (*mcr-1* to *mcr-10*) have been reported all over the world since the first report of *mcr-1* in 2016 (1, 2). However, the *mcr-1* gene is still the most widespread of the *mcr* genes and has been identified on both broad-host-range and narrow-host-range plasmids of different replicons, including IncX3, IncX4, IncH1, IncHI1, IncHI2, IncP, IncI2, IncF, IncFII, and IncY (3–6). Resistance to the last-line antibiotic colistin mediated by *mcr-1* represents a threat to global public health. Strains positive for *mcr-1* in various sources have been reported. Père David’s deer is a highly endangered species originating in China, and many deer are currently being raised in nature reserve settings. Dissemination of antimicrobial resistance (AMR) bacteria among animals poses a potential threat to the environment. Therefore, research on the dissemination of *mcr-1*-positive Escherichia coli from Père David’s deer is of great significance. To our knowledge, the distribution of *mcr-1* in Père David’s Deer National Nature Reserve remained to be investigated up to now. To further learn the transmission characteristics of *mcr-1* among nature reserve sites, we studied the prevalence of *mcr-1* in E. coli from Père David’s deer and proved that *mcr-1* has been widespread in the nature reserve.

Among 97 samples, 55 (56.70%) yielded 67 *mcr-1*-positive strains, and no other *mcr* variants were found. All the *mcr-1*-positive strains were identified as E. coli. This indicated that *mcr-1*-positive E. coli strains existed in nature reserve environments at a high prevalence. All 67 *mcr-1*-positive isolates conferred resistance to colistin (MICs ranging from 4 to 8 mg/liter) (see Table S1 in the supplemental material). The antibiotic resistance rates of β-lactams amoxicillin, aztreonam, and ceftiofur were very high. We further identified genes encoding β-lactamases through multiplex PCR methods, as previously reported (7). There were 65 (97.01%) *mcr-1*-positive isolates containing β-lactamase genes (Tables S2 and S3), with *bla*_CTX-M_ (57 strains) and *bla*_TEM_ (19 strains) most widely distributed in these *mcr-1*-positive isolates (Tables S2 and S3). *bla*_CTX-M_ and *bla*_TEM_ were prevalent extended-spectrum-β-lactamase (ESBL) genes conferring resistance to most β-lactam antibiotics (8). In addition, genes encoding OXA-1-like broad-spectrum β-lactamases were detected in 7 strains harboring IncX4 type plasmids (Table S2). The *mcr-1* gene from 66 of 67 isolates and the resistance phenotype were successfully transferred to E. coli J53 (Table S1), suggesting that the *mcr-1* gene was located in conjugative plasmids or other mobilizable genetic elements (MGEs) in the 66 isolates. PCR-based replicon typing (PBRT) was performed for all 67 *mcr-1*-positive isolates and 66 transconjugants. Results showed that 44 transconjugants harbored only an IncI2 plasmid, but corresponding parental strains contained 1 to 4 replicon types, including IncI2. Eight transconjugants harbored only an IncX4 plasmid, and corresponding parental strains also had only an IncX4 plasmid (Table S2). It was concluded that *mcr-1* was located in IncI2 or IncX4 plasmids in these strains. Two replicon types of IncHI2 and IncN from 13 transconjugants were identified, and 2 to 4 replicon types, including IncHI2 and IncN, were detected from their corresponding parental strains. It has been reported that the *mcr-1*-positive IncHI2 plasmid pMCR1_1943 (265,538 bp) also had an IncN replicon (9). S1 nuclease-based pulsed-field gel electrophoresis (S1-PFGE) of 8 transconjugants showed that only one plasmid was visible with a size similar to pMCR1_1943. Therefore, we considered that *mcr-1* was located in the IncHI2 plasmid in the 13 strains. However, there were no replicon types detected in strain LD27-1, which implied a possible chromosomal location of *mcr-1*. In addition, replicon types of one transconjugant were exactly the same as those of its parental strain LD91-1, with four replicon types, IncHI2, IncN, IncFIB, and IncF, detected simultaneously. According to the plasmid size in the S1-PFGE fingerprint of TLD91-1 (transconjugant of LD91-1), we speculated that the plasmid of TLD91-1 was a fused plasmid formed during conjugation whose molecular mechanism was reported previously (10).

10.1128/mSphere.01221-20.3 TABLE S1 Antibiotic susceptibility profiles (mg/liter) of 67 E. coli strains that harbor the *mcr-1* gene. Download Table S1, DOCX file, 0.02 MB. Copyright © 2020 Lu et al. 2020 Lu et al. This content is distributed under the terms of the Creative Commons Attribution 4.0 International license.

10.1128/mSphere.01221-20.4 TABLE S2 Replicon types and β-lactamase genes of 67 *mcr-1*-positive E. coli strains and their transconjugants. Download Table S2, DOCX file, 0.02 MB. Copyright © 2020 Lu et al. 2020 Lu et al. This content is distributed under the terms of the Creative Commons Attribution 4.0 International license.

10.1128/mSphere.01221-20.5 TABLE S3 Positive rate of β-lactamase genes in 67 *mcr-1*-positive E. coli strains. Download Table S3, DOCX file, 0.01 MB. Copyright © 2020 Lu et al. 2020 Lu et al. This content is distributed under the terms of the Creative Commons Attribution 4.0 International license.

PFGE patterns with a cutoff at 90% similarity were considered to belong to the same phylogenetic cluster and were indicated as groups A to W, implying diverse strain clones. Two different *mcr-1*-positive strains with diverse PFGE types were detected in the same sample among 12 samples according to their colony difference in morphology and color (Table 1; Fig. S1). A single sample containing multiple *mcr-1*-positive isolates suggested that *mcr-1* had spread in the same microbiota. We also found that the *mcr-1*-harboring plasmids carried by these strains were diverse. IncX4 and IncI2 type *mcr-1*-bearing plasmids appeared in two strains, respectively, in samples LD4 and LD9, indicating that coexistence of different *mcr-1*-bearing plasmids in diverse bacteria of the same microbiota sample would exacerbate the transmission of resistance genes. In samples LD26, LD39, LD54, LD75, and LD91, *mcr-1*-positive plasmids with IncHI2 and IncI2 types were found simultaneously in two different strains. Ten strains from samples LD24, LD36, LD38, LD70, and LD94 carried *mcr-1*-positive IncI2 plasmids (Table 1). In these samples, strains with the extension “-2” were distributed mainly in two PFGE types (11 strains belonging to group A and 1 strain belonging to group F), while the strains with names ending in “-1” were classified into 8 PFGE types (B, C, E, G, H, K, L, and S). This result indicates that the samples are highly diverse. Partial *mcr-1*-positive strains isolated from Père David’s deer were shown to exhibit genetically similar PFGE types, implying that clonal spread occurred in the nature reserve. *mcr-1*-positive plasmids with the same replicon type were found in different PFGE clusters, indicating that horizontal dissemination of the *mcr-1* gene by plasmids also existed (Fig. S1).

**TABLE 1:** Basic information about 12 samples detected with two *mcr-1*-positive strains

Sample name	Strain designation		Replicon type(s)	
	Parental strain	Transconjugant	Parental strain	Transconjugant
LD4	LD4-1	TLD4-1	IncX4	IncX4
	LD4-2	TLD4-2	IncI2	IncI2
LD9	LD9-1	TLD9-1	IncX4	IncX4
	LD9-2	TLD9-2	IncI2	IncI2
LD24	LD24-1	TLD24-1	IncHI2/IncF/IncFIB/IncI2	IncI2
	LD24-2	TLD24-2	IncI2	IncI2
LD26	LD26-1	TLD26-1	IncHI2/IncN/IncFIB/IncF	IncHI2/IncN
	LD26-2	TLD26-2	IncI2	IncI2
LD36	LD36-1	TLD36-1	IncHI2/IncF/IncFIB/IncI2	IncI2
	LD36-2	TLD36-2	IncI2	IncI2
LD38	LD38-1	TLD38-1	IncHI1/IncI2/IncFIB	IncI2
	LD38-2	TLD38-2	IncI2	IncI2
LD39	LD39-1	TLD39-1	IncHI2/IncN/IncFIA/IncFIB	IncHI2/IncN
	LD39-2	TLD39-2	IncI2	IncI2
LD54	LD54-1	TLD54-1	IncHI2/IncN	IncHI2/IncN
	LD54-2	TLD54-2	IncI2	IncI2
LD70	LD70-1	TLD70-1	IncI2	IncI2
	LD70-2	TLD70-2	IncI2	IncI2
LD75	LD75-1	TLD75-1	IncHI2/IncN/IncFIB/IncF	IncHI2/IncN
	LD75-2	TLD75-2	IncI2	IncI2
LD91	LD91-1	TLD91-1	IncHI2/IncN/IncFIB/IncF	IncHI2/IncN
	LD91-2	TLD91-2	IncI2/IncFIB	IncI2
LD94	LD94-1	TLD94-1	IncI2	IncI2
	LD94-2	TLD94-2	IncI2	IncI2

10.1128/mSphere.01221-20.1 FIG S1 PFGE-XbaI dendrogram of *mcr-1*-positive *E. coli* isolates. The PFGE assay was conducted according to the standard protocol. PFGE patterns with a cutoff at 90% similarity (indicated by a dashed line) were considered to belong to the same PFGE cluster and are indicated as groups A to W. The last column indicates replicon types of *mcr-1*-bearing plasmids. Download FIG S1, JPG file, 0.2 MB. Copyright © 2020 Lu et al. 2020 Lu et al. This content is distributed under the terms of the Creative Commons Attribution 4.0 International license.

To obtain a comprehensive view of the genetic features of *mcr-1*-bearing MGEs in these isolates, five representative *mcr-1*-positive E. coli strains were selected for further genome characterization. Whole-genome sequencing (WGS) and bioinformatics analyses showed that isolate LD27-1 harbored a chromosome (sequence type 10 [ST10]) 4,694,065 bp in length (Table 2), with *mcr-1* located in the chromosome. A 9,974-bp chromosomal segment containing *mcr-1* was extracted, and BLASTn analysis was performed, showing that the segment had 99.98% identity (100% coverage) to the sequence of the E. coli L73 chromosome (CP033378) isolated from goose and 99.99% identity (100% coverage) to the sequence of the E. coli PE15 chromosome (CP041628) of pig origin. A hypothetical protein was disrupted by insertion of IS*Apl1*-*mcr-1*-*orf*, which may derive from Tn*6330* (IS*Apl1*-*mcr-1*-*orf-*IS*Apl1*) that L73 and PE15 chromosomes contained (3) (Fig. S2).

**TABLE 2:** Detailed information about plasmids of five sequenced *mcr-1*-positive strains

Strains	Sequence type	Plasmid	Accession no.	Size (bp)	Replicon type(s) (accession no.)	Resistance gene(s)
LD26-1	ST3714	pLD26-1-MCR1	CP047666	251,000	IncHI2, IncHI2A, IncN	*mph*(A), *aac(3)-IV*, *aadA1*, *aadA2*, *aph(3')-Ia*, *aph(4)-Ia*, *fosA3*, *dfrA12*, *mcr-1*, *sul1*, *sul2*, *sul3*, *bla*_CTX-M-14_, *cmlA1*, *floR*
		pLD26-1-135kb	CP047667	135,123	IncFIB (AP001918), IncFII	*dfrA14*, *tet*(A), *floR*, *bla*_TEM-135_, *qnrS1*
LD22-1	ST3714	pLD22-1- MCR1	CP047877	251,000	IncHI2, IncHI2A, IncN	*bla*_CTX-M-14_, *mph*(A), *mcr-1*, *aac(3)-IV*, *aadA1*, *aadA2*, *aph(3‘)-Ia*, *aph(4)-Ia*, *cmlA1*, *floR*, *fosA3*, *sul1*, *sul2*, *sul3*, *dfrA12*
		pLD22-1-135kb	CP047878	135,123	IncFIB (AP001918), IncFII	*dfrA14*, *tet*(A), *floR*, *bla*_TEM-135_, *qnrS1*
		pLD22-1-6kb	CP047879	6,430	None	None
LD39-1	ST2325	pLD39-1- MCR1	CP047659	251,000	IncHI2, IncHI2A, IncN	*bla*_CTX-M-14_, *fosA3*, *mph*(A), *cmlA1*, *floR*, *mcr-1*, *aac(3)-IV*, *aadA1*, *aadA2*, *aph(3')-Ia*, *aph(4)-Ia*, *dfrA12*, *sul1*, *sul2*, *sul3*
		pLD39-1-134kb	CP047660	134,831	IncFIB (pB171), IncFIA	None
		pLD39-1-6kb	CP047661	6,938	None	None
LD67-1	ST1485	pLD67-1-MCR1	CP061186	66,568	IncI2	*mcr-1*, *bla*_CTX-M-132_
		pLD67-1-157kb	CP061187	157,028	IncHI2, IncHI2A	*aadA1*, *dfrA14*, *tet*(A), *qnrS1*, *ARR-2*, *bla*_OXA-10_, *cmlA1*, *floR*
		pLD67-1-165kb	CP061188	165,427	IncFIA, IncFIB (AP001918)	*dfrA14*, *bla*_TEM-1B_, *aph(3'')-Ib*, *aph(6)-Id*, *sul2*
LD93-1	ST7511	pLD93-1-90kb	CP047663	90,674	None	None
		pLD93-1-MCR1	CP047664	33,309	IncX4	*mcr-1*

10.1128/mSphere.01221-20.2 FIG S2 Linear sequence alignment between the selected fragment of E. coli LD27-1 chromosome and E. coli L73 and PE15 chromosome fragments. Download FIG S2, JPG file, 0.1 MB. Copyright © 2020 Lu et al. 2020 Lu et al. This content is distributed under the terms of the Creative Commons Attribution 4.0 International license.

**FIG 1:**
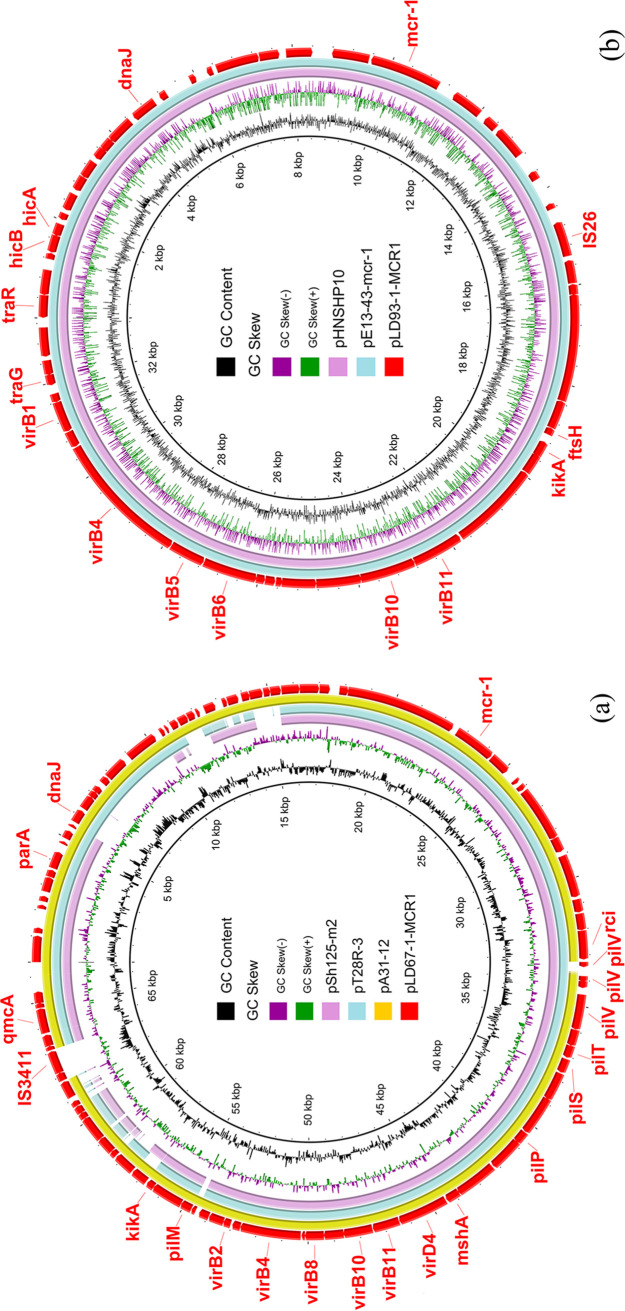

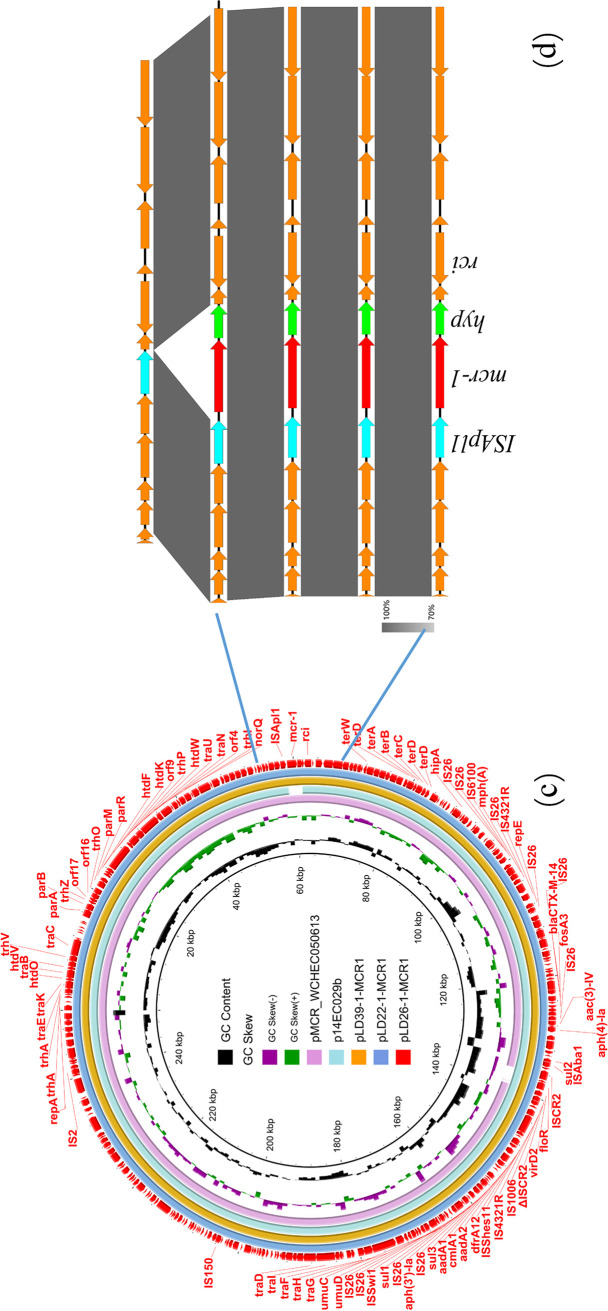
Sequence comparison of plasmids harboring the *mcr-1* gene. (a) Circular comparison between *mcr-1*-bearing IncI2 plasmid pLD67-1-MCR1 in strain LD67-1 in this study and three similar IncI2 plasmids in the NCBI nr database. pLD67-1-MCR1 was used as the reference in the outermost ring. (b) Circular comparison between *mcr-1*-carrying IncX4 plasmid pLD93-1-MCR1 in strain LD93-1 in this study and two similar IncX4 plasmids in the NCBI nr database. The outer circle with red arrows denotes annotation of reference plasmid pLD93-1-MCR1. (c) Circular comparison between three *mcr-1*-bearing IncHI2 plasmids and two similar IncHI2 plasmids in the NCBI nr database. (d) Linear comparison of the partial sequence containing *mcr-1* of IncHI2 type plasmids (pLD22-1, pLD26-1, and pLD39-1) with two similar structures of plasmids pMCR_WCHEC050613 and p14EC029b in the NCBI nr database.

LD67-1 harbored a chromosome (ST1485) and three plasmids consisting of pLD67-1-MCR1 (66,568 bp), pLD67-1-157kb (157,028 bp), and pLD67-1-165kb (165,427 bp). pLD67-1-MCR1 was an *mcr-1*-bearing IncI2 plasmid carrying the ESBL gene *bla*_CTX-M-132_ and with an overall 99.14% nucleotide identity and 100% query coverage to the sequence of E. coli A31-12 plasmid pA31-12 (GenBank accession no. KX034083) (Fig. 1a), which was an *mcr-1*-harboring plasmid with *bla*_CTX-M-55_. pA31-12 was the first IncI2 plasmid coharboring *bla*_CTX-M-55_ and *mcr-1* (11). pLD67-1-MCR1 showed 99.61% identity (90% coverage) to E. coli T28R plasmid pT28R-3 (CP049356) (Fig. 1a), which was an *mcr-1-* and *bla*_CTX-M-64_-carrying plasmid. No other CTX-M genes were found in *mcr-1*-carrying IncI2 plasmid in the nr/nt database (as of 1 September 2020). We first reported the IncI2 plasmid harboring both *bla*_CTX-M-132_ and *mcr-1* in this study. pLD67-1-MCR1 showed 99.79% identity (82% coverage) with Shigella sonnei SH11Sh125 *mcr-1*-carrying plasmid pSh125-m2 (KY363998) (Fig. 1a) without CTX-M genes. Cotransfer of *mcr-1* with *bla*_CTX-M-132_ by a single conjugative plasmid constitutes a serious threat. pLD67-1-157kb (157,028 bp) was an IncHI2 plasmid with resistance genes *aadA1*, *dfrA14*, *tet*(A), *qnrS1*, *ARR-2*, *bla*_OXA-10_, *cmlA1*, and *floR* (Table 2). pLD67-1-165kb (165,427 bp) was an IncFIA and IncFIB multidrug resistance plasmid carrying *dfrA14*, *bla*_TEM-1B_, *aph(3′')-Ib*, *aph(6)-Id*, and *sul2* (Table 2).

One chromosome and two plasmids, pLD93-1-90kb (90,674 bp) and pLD93-1-MCR1 (33,309 bp), were found in strain LD93-1, which belonged to ST7511. The *mcr-1*-positive plasmid pLD93-1-MCR1 was an IncX4 plasmid sharing 100% identity (100% coverage) to pE13-43-*mcr-1* (GenBank accession no. MG747473) (Fig. 1b) from human urine. pLD93-1-MCR1 also showed 100% identity and 100% coverage to pHNSHP10 (MF774182) (Fig. 1b) of pig origin, indicating that *mcr-1*-bearing IncX4 plasmids could disseminate in human, food animals, and Pere David’s deer. There were no plasmid replicons and resistance genes found in pLD93-1-90kb (Table 2).

Three *mcr-1*-bearing IncHI2-positive strains, LD22-1, LD26-1, and LD39-1, were randomly selected to perform genomic analysis. LD22-1 and LD26-1 belonged to ST3714, and LD39-1 belonged to ST2325. They possessed the typical IncHI2 *mcr-1*-bearing plasmids pLD22-1-MCR1, pLD26-1-MCR1, and pLD39-1-MCR1, sharing 100% identity with each other (251,000 bp in length) and carried the same resistance genes, namely, *bla*_CTX-M-14_, *fosA3*, *mph*(A), *cmlA1*, *floR*, *aac(3)-IV*, *aadA1*, *aadA2*, *aph(3′)-Ia*, *aph(4)-Ia*, *dfrA12*, *sul1*, *sul2*, and *sul3* (Table 2). BLASTn analysis showed that pLD26-1-MCR1 exhibited 99.98% identity (98% coverage) to plasmid pMCR_WCHEC050613 (GenBank accession no. CP019214) and 99.99% identity (98% coverage) to plasmid p14EC029b (CP024143) (Fig. 1c). IS*Apl1* upstream of *mcr-1* played a crucial part in the presence of *mcr-1* (Fig. 1d). IncFIB-IncFII plasmid pLD22-1-135kb (135,123 bp) showed 100% identity to pLD26-1-135kb (135,123 bp), carrying resistance genes *dfrA14*, *tet*(A), *floR*, *bla*_TEM-135_, and *qnrS1*. pLD39-1-134kb (134,831 bp) was an IncFIA-IncFIB plasmid and had no resistance genes. Small plasmids pLD22-1-6kb and pLD39-1-6kb were found in strain LD22-1 and LD39-1 (Table 2).

Our investigation found that there is a supplementary feeding area in the nature reserve where commercial feed additives such as colistin may have been used in feedstuff before May 2017. Widespread *mcr-1* dissemination in Père David’s deer may be attributable to selective pressure exerted by colistin. It has been suggested that *mcr-1* spreads to humans from farmed animals (1, 12). As a result, China banned the use of colistin as a feed additive for animals on 1 May 2017 (13). It has been reported that the *mcr-1* prevalence decreased significantly in national pig farms after the ban of colistin in animal feed (14). A reduction in the *mcr-1*-positive E. coli population size following the colistin ban could also be expected in Père David’s deer, which warrants further surveillance.

The global spread of ESBL producers is of great concern to human and animal health, and CTX-Ms are the most predominant ESBLs worldwide (15). CTX-M-producing E. coli isolates were recognized as a major cause of hospital- and community-onset infections (16, 17). The coexistence of ESBL genes and *mcr-1* in E. coli with multidrug resistance was first reported in China in 2016 (18). Evidence showed that ESBL-producing E. coli was more likely to recruit the *mcr-1* gene than non-ESBL-producing E. coli (19). Given the fact that colistin is one of the last-line antibiotics for managing multidrug-resistant infections, cotransfer of *mcr-1* with ESBL genes by a single mobile plasmid might compromise clinical treatment considerably. In our work, a high prevalence of IncI2 and IncHI2 plasmids harboring both *bla*_CTX-M_ and *mcr-1* (Table 2; Table S2) indicates a widespread situation of such notorious MDR plasmids among animals. The emergence of the IncI2 plasmid harboring *bla*_CTX-M-132_ and *mcr-1* reported here should arouse our attention.

The limitations of this study are that geographical distribution and the number of samples collected are relatively confined. Although we collected fresh fecal samples from areas as scattered as possible to guarantee that fecal samples were derived from different individuals of Pere David's deer, duplicate sampling of the same animal was possible.

To conclude, this research is the first report of the prevalence of colistin resistance gene *mcr-1* in E. coli from Père David’s deer in China that was mainly mediated by plasmids. Mobilizable *mcr-1*-positive plasmids (IncI2, IncX4, and IncHI2) were widespread in Père David’s Deer National Nature Reserve, which may be an important reservoir of *mcr-1*. The IncI2 *mcr-1*-bearing plasmid was the most frequent plasmid type (44/67, 65.67%) in the nature reserve, and we are the first to report the IncI2 plasmid harboring both *bla*_CTX-M-132_ and *mcr-1*. Wide spread of *mcr-1-*harboring IncHI2 plasmids carrying multiple resistance genes in the nature reserve constitutes an easily missed source of multidrug-resistant Gram-negative bacteria. The horizontal dissemination of the *mcr-1* gene by plasmids needs to be further investigated, and monitoring of other important emerging resistance genes in nature reserves of animals should be performed.

### Bacterial isolates and identification.

On 18 August 2018, a total of 97 fresh fecal samples were collected from Père David’s Deer National Nature Reserve in Dafeng, Jiangsu Province, China. Individual fresh fecal samples were collected using a sterile swab, subsequently diluted in brain heart infusion broth containing 2 mg/liter colistin, and incubated for 6 h at 37°C. The cultures were plated on MacConkey agar and incubated for 12 h at 37°C. One to three colonies with different morphological characteristics from each MacConkey agar plate were purified and subsequently screened for *mcr-1* to *mcr-5* genes by multiplex PCR method as previously reported (20). 16S rRNA gene sequencing was performed to confirm bacterial species.

### Antimicrobial susceptibility testing.

The MICs of colistin (CST), aztreonam (ATM), amoxicillin (AMC), florfenicol (FFC), ceftiofur (CFF), streptomycin (STR), doxycycline (DOX), meropenem (MEM), and enrofloxacin (ENR) for all *mcr-1*-bearing isolates were determined by the broth microdilution method and interpreted in accordance with the Clinical and Laboratory Standards Institute (CLSI) guidelines (21) and the European Committee on Antimicrobial Susceptibility Testing (EUCAST) breakpoints (https://www.eucast.org/clinical_breakpoints/). Colistin was interpreted in accordance with the EUCAST (susceptible, ≤2 mg/liter; resistant, >2 mg/liter). Reference strain E. coli ATCC 25922 served as the quality control strain.

### Conjugation experiments and plasmid replicon typing.

To investigate the transferability of *mcr*-carrying plasmids, conjugation assays were performed using MCR-producing strains as donors and E. coli J53 (Azi^r^) as the recipient. Bacterial strains were streaked onto LB agar plates, followed by inoculation into LB broth overnight. Cultures of donors and the recipient were mixed 1:3, and then 100 μl of mixed culture was applied onto LB agar plates, followed by incubation at 37°C for 16 to 20 h. After incubation, we subsequently diluted the mixed culture on LB agar plates in sterile saline. LB agar plates supplemented with colistin (2 mg/liter) and sodium azide (150 mg/liter) were used to recover transconjugants. The presence of *mcr* genes in transconjugants was confirmed by PCR and antimicrobial susceptibility testing as described above. The plasmid contents of *mcr*-positive strains and transconjugants were determined by PBRT (22) with the addition of PCR detections of IncX4 and IncI2 plasmids, as previously described (3).

### XbaI-PFGE and S1-PFGE.

XbaI pulsed-field gel electrophoresis (PFGE) was performed to assess the genetic relatedness of all *mcr*-positive isolates. Briefly, the whole-cell DNA of E. coli isolates was digested with the XbaI restriction enzyme for 3 h at 37°C. Electrophoresis was conducted on a CHEF-DR III apparatus (Bio-Rad, Hercules, CA, USA) through a 1% agarose gel in 0.5× Tris-borate-EDTA buffer using an initial pulse time of 4 s and a final pulse time of 45 s at a voltage of 200 V for 20 h at 14°C (23). Genomic DNA of the Salmonella enterica serovar Braenderup strain H9812 restricted with XbaI (TaKaRa, Osaka, Japan) was used as the reference standard. Cluster analysis of XbaI-PFGE fingerprints was typically performed by using BioNumerics 7.6 (Applied Maths, Sint-Martens-Latem, Belgium). Bacterial DNA was prepared in agarose plugs and digested with 1 U of S1 nuclease (New England Biolabs) for S1 nuclease-based PFGE (S1-PFGE).

### Plasmid sequencing and bioinformatics analyses.

The genomic DNA of several *mcr-1*-positive E. coli strains was extracted using a TIANamp bacteria DNA kit (Tiangen, Chain) in accordance with the manufacturer’s recommendation. Whole-genome sequencing was performed via Illumina HiSeq and Oxford Nanopore Technologies (ONT) MinION platforms. *De novo* assembly was performed by Unicycler, combining short-read and long-read data (24). The Rapid Annotation using Subsystems Technology annotation website server (https://rast.nmpdr.org/rast.cgi) was then used to annotate the genomes (25). Online tools including PlasmidFinder 2.1, ResFinder 3.2, and MLST 2.0 (multilocus sequence typing) were utilized to assemble and characterize the *mcr-1*-bearing genomes (https://cge.cbs.dtu.dk/services/). Comparisons with highly homologous complete plasmid sequences available in NCBI for the plasmids in the study were performed with BRIG (26). To visualize the genetic comparison features, Easyfig was used to generate linear figures (27).

### Data availability.

The complete sequences of LD22-1, LD26-1, LD27-1, LD39-1, LD67-1, and LD93-1 were deposited in the NCBI database under the following accession numbers: LD22-1 chromosome, CP047876; LD26-1 chromosome, CP047665; LD27-1 chromosome, CP047594; LD39-1 chromosome, CP047658; LD67-1 chromosome, CP061185; and LD93-1 chromosome, CP047662. Accession numbers of all plasmids are listed in Table 2.
