# Supplementary material for: Widespread Prevalence of Plasmid-Mediated Colistin Resistance Gene mcr-1 in Escherichia coli from Père David's Deer in China
Source: mSphere. 2020 Dec 23;5(6):e01221-20. doi: 10.1128/mSphere.01221-20 (PMC7763555; doi:10.1128/mSphere.01221-20)
Supplement: TABLE S1 [file mSphere.01221-20-st001.docx]

| Strain ID | | CST | | FFC | | CFF | | DOX | | ATM | | STR | | AMC | | ENR | | MEM | | NaN3 | |
| --- | --- | --- | --- | --- | --- | --- | --- | --- | --- | --- | --- | --- | --- | --- | --- | --- | --- | --- | --- | --- | --- |
| Partental strains | Transconjugants | P | T | P | T | P | T | P | T | P | T | P | T | P | T | P | T | P | T | P | T |
| LD3-1 | TLD3-1 | 4 | 4 | >64 | 8 | 1 | 1 | 16 | 2 | ≤0.125 | 0.5 | 128 | 0.5 | 128 | 8 | 0.03 | 0.008 | ≤0.004 | 0.015 | 128 | 1280 |
| LD4-1 | TLD4-1 | 4 | 4 | >64 | 4 | 1 | 1 | 32 | 2 | ≤0.125 | ≤0.125 | 32 | 1 | 128 | 4 | 0.03 | 0.008 | ≤0.004 | 0.015 | 128 | 1280 |
| LD4-2 | TLD4-2 | 4 | 4 | 4 | 4 | >64 | >64 | 1 | 2 | 64 | 64 | 8 | 2 | >128 | >128 | 0.03 | 0.008 | ≤0.004 | 0.015 | 128 | 1280 |
| LD5-1 | TLD5-1 | 4 | 4 | 4 | 4 | >64 | >64 | 8 | 2 | 64 | 64 | 8 | 2 | >128 | >128 | 0.008 | 0.008 | ≤0.004 | 0.015 | 256 | 1280 |
| LD7-2 | TLD7-2 | 4 | 4 | >64 | >64 | >64 | 64 | 2 | 2 | 1 | 2 | 32 | 8 | >128 | >128 | 0.008 | 0.008 | ≤0.004 | 0.015 | 128 | 1280 |
| LD8-1 | TLD8-1 | 4 | 4 | 4 | 4 | >64 | >64 | 8 | 2 | 64 | >64 | 4 | 2 | >128 | >128 | 0.008 | 0.008 | 0.008 | 0.015 | 128 | 1280 |
| LD9-1 | TLD9-1 | 4 | 4 | >64 | 8 | 1 | 1 | 16 | 4 | 0.06 | 0.25 | 64 | 4 | 128 | 8 | 0.015 | 0.008 | ≤0.004 | 0.008 | 128 | 1280 |
| LD9-2 | TLD9-2 | 8 | 4 | 8 | 8 | >64 | >64 | 1 | 2 | >64 | >64 | 16 | 0.5 | 128 | >128 | 0.008 | 0.008 | 0.008 | 0.015 | 64 | 1280 |
| LD13-1 | TLD13-1 | 4 | 4 | >64 | 8 | 1 | 0.5 | 8 | 2 | 0.06 | ≤0.125 | 16 | 0.5 | 128 | 8 | 0.03 | 0.008 | ≤0.004 | 0.015 | 64 | 1280 |
| LD14-1 | TLD14-1 | 4 | 4 | 4 | 8 | 0.5 | 1 | 32 | 2 | ≤0.125 | ≤0.125 | 4 | 0.25 | 4 | 4 | 0.5 | 0.008 | ≤0.004 | 0.015 | 64 | 1280 |
| LD16-1 | TLD16-1 | 8 | 4 | >64 | 8 | 1 | 1 | 16 | 2 | 1 | 0.25 | 16 | 0.5 | 128 | 8 | 0.03 | 0.008 | ≤0.004 | 0.015 | 128 | 1280 |
| LD18-1 | TLD18-1 | 4 | 4 | 4 | 4 | >64 | >64 | 1 | 2 | >64 | 64 | 8 | 0.5 | >128 | >128 | 0.008 | 0.008 | 0.008 | 0.015 | 128 | 1280 |
| LD20-1 | TLD20-1 | 4 | 4 | >64 | 4 | >64 | >64 | 16 | 2 | 64 | 32 | >128 | 0.5 | >128 | >128 | 0.5 | 0.008 | 0.008 | 0.015 | 128 | 1280 |
| LD22-1 | TLD22-1 | 4 | 4 | >64 | >64 | >64 | 64 | 16 | 2 | 1 | 2 | 16 | 8 | >128 | >128 | 4 | 0.008 | 0.008 | 0.015 | 64 | 1280 |
| LD24-1 | TLD24-1 | 8 | 4 | >64 | 4 | >64 | >64 | 8 | 2 | 64 | >64 | >128 | 4 | >128 | >128 | 0.5 | 0.008 | 0.008 | 0.008 | 32 | 1280 |
| LD24-2 | TLD24-2 | 4 | 4 | >64 | 8 | 2 | 1 | 8 | 2 | 0.25 | 0.5 | 64 | 0.25 | >128 | 4 | 0.5 | 0.008 | 0.008 | 0.015 | 64 | 1280 |
| LD25-1 | TLD25-1 | 4 | 4 | 8 | 4 | >64 | >64 | 2 | 1 | 64 | 64 | 8 | 0.5 | >128 | >128 | 0.015 | 0.008 | 0.015 | 0.008 | 64 | 1280 |
| LD26-1 | TLD26-1 | 4 | 4 | >64 | >64 | >64 | 64 | 16 | 4 | 2 | 2 | 16 | 2 | >128 | >128 | 8 | 0.008 | ≤0.004 | 0.008 | 128 | 1280 |
| LD26-2 | TLD26-2 | 4 | 4 | 8 | 4 | >64 | >64 | 4 | 2 | >64 | 64 | 16 | 4 | >128 | >128 | 0.008 | 0.004 | ≤0.004 | 0.008 | 64 | 1280 |
| LD27-1 | - | 8 | - | 8 | - | 1 | - | 64 | - | ≤0.125 | - | 32 | - | 2 | - | 0.125 | - | 0.008 | - | 128 | - |
| LD28-1 | TLD28-1 | 4 | 4 | >64 | 64 | >64 | 64 | 4 | 2 | 1 | 2 | 16 | 4 | >128 | >128 | 0.015 | 0.008 | 0.015 | 0.015 | 128 | 1280 |
| LD31-2 | TLD31-2 | 4 | 4 | 4 | 8 | >64 | >64 | 2 | 2 | 32 | 64 | 16 | 4 | >128 | >128 | 0.015 | 0.008 | 0.008 | 0.008 | 128 | 1280 |
| LD36-1 | TLD36-1 | 4 | 4 | >64 | 4 | >64 | >64 | 16 | 2 | >64 | >64 | >128 | 2 | >128 | >128 | 0.5 | 0.008 | 0.008 | 0.015 | 64 | 1280 |
| LD36-2 | TLD36-2 | 4 | 4 | 8 | 4 | >64 | >64 | 2 | 2 | 64 | >64 | 16 | 4 | >128 | >128 | 0.008 | 0.004 | ≤0.004 | 0.015 | 128 | 1280 |
| LD37-1 | TLD37-1 | 4 | 4 | 4 | 8 | 0.5 | 1 | 16 | 2 | ≤0.125 | ≤0.125 | 8 | 2 | >128 | 4 | 0.5 | 0.008 | 0.008 | 0.015 | 64 | 1280 |
| LD38-1 | TLD38-1 | 4 | 4 | 4 | 4 | 64 | >64 | 128 | 2 | >64 | >64 | 16 | 2 | >128 | >128 | 4 | 0.008 | ≤0.004 | 0.015 | 64 | 1280 |
| LD38-2 | TLD38-2 | 4 | 4 | 4 | 4 | >64 | >64 | 4 | 2 | 64 | 64 | 8 | 2 | >128 | >128 | 0.015 | 0.008 | ≤0.004 | 0.03 | 128 | 1280 |
| LD39-1 | TLD39-1 | 4 | 4 | >64 | >64 | 32 | 32 | 2 | 1 | 1 | 1 | 16 | 8 | >128 | >128 | 0.015 | 0.015 | 0.008 | 0.008 | 64 | 1280 |
| LD39-2 | TLD39-2 | 8 | 4 | 4 | 8 | >64 | >64 | 2 | 1 | 64 | >64 | 4 | 1 | >128 | >128 | 0.015 | 0.008 | 0.015 | 0.015 | 128 | 1280 |
| LD40-1 | TLD40-1 | 4 | 4 | >64 | 4 | >64 | >64 | 8 | 1 | 64 | >64 | >128 | 2 | >128 | >128 | 1 | 0.015 | 0.008 | 0.008 | 128 | 1280 |
| LD41-1 | TLD41-1 | 4 | 4 | >64 | 4 | >64 | >64 | 8 | 1 | 64 | 64 | >128 | 0.5 | >128 | >128 | 1 | 0.015 | 0.015 | 0.015 | 128 | 1280 |
| LD42-1 | TLD42-1 | 4 | 4 | >64 | 8 | >64 | >64 | 8 | 2 | 32 | 64 | 128 | 1 | >128 | >128 | 1 | 0.008 | 0.008 | 0.015 | 64 | 1280 |
| LD47-2 | TLD47-2 | 4 | 4 | 4 | 4 | >64 | >64 | 1 | 2 | 64 | 32 | 16 | 2 | >128 | >128 | 0.015 | 0.008 | 0.008 | 0.008 | 128 | 1280 |
| LD48-1 | TLD48-1 | 4 | 4 | 4 | 4 | 1 | 1 | 16 | 2 | ≤0.125 | ≤0.125 | 128 | 2 | >128 | 4 | 16 | 0.008 | 0.008 | 0.015 | 64 | 1280 |
| LD50-1 | TLD50-1 | 4 | 4 | >64 | >64 | >64 | 64 | 2 | 2 | 1 | 2 | 32 | 2 | >128 | >128 | 0.03 | 0.008 | 0.008 | 0.015 | 64 | 1280 |
| LD51-1 | TLD51-1 | 4 | 4 | 4 | 8 | >64 | >64 | 1 | 2 | >64 | 64 | 8 | 0.25 | >128 | >128 | 0.008 | 0.008 | ≤0.004 | 0.015 | 128 | 1280 |
| LD52-1 | TLD52-1 | 4 | 4 | 8 | 4 | 1 | 1 | 2 | 2 | ≤0.125 | 0.25 | 2 | 8 | 4 | 8 | 0.03 | 0.004 | 0.008 | 0.008 | 128 | 1280 |
| LD53-2 | TLD53-2 | 4 | 4 | 4 | 4 | >64 | >64 | 2 | 2 | 32 | 64 | 8 | 0.5 | >128 | >128 | 0.03 | 0.008 | 0.008 | 0.015 | 64 | 1280 |
| LD54-1 | TLD54-1 | 4 | 4 | >64 | >64 | >64 | 64 | 1 | 2 | 2 | 2 | 16 | 4 | >128 | >128 | 0.008 | 0.008 | ≤0.004 | 0.008 | 64 | 1280 |
| LD54-2 | TLD54-2 | 4 | 4 | 4 | 4 | >64 | >64 | 4 | 2 | >64 | >64 | 8 | 1 | >128 | >128 | 0.015 | 0.008 | ≤0.004 | 0.015 | 128 | 1280 |
| LD55-2 | TLD55-2 | 4 | 4 | >64 | 8 | 0.5 | 0.5 | 16 | 1 | ≤0.125 | ≤0.125 | 32 | 0.5 | >128 | 4 | 0.5 | 0.008 | 0.008 | 0.008 | 64 | 1280 |
| LD65-1 | TLD65-1 | 4 | 4 | 4 | 8 | 1 | 1 | 16 | 2 | ≤0.125 | 0.25 | 16 | 2 | >128 | 8 | 2 | 0.008 | 0.008 | 0.015 | 64 | 1280 |
| LD67-1 | TLD67-1 | 4 | 4 | >64 | 8 | >64 | >64 | 8 | 2 | 64 | >64 | >128 | 0.25 | >128 | >128 | 1 | 0.008 | 0.03 | 0.015 | 128 | 1280 |
| LD68-2 | TLD68-2 | 4 | 4 | 4 | 4 | >64 | >64 | 1 | 2 | 32 | >64 | 16 | 0.5 | >128 | >128 | ≤0.004 | 0.008 | 0.03 | 0.015 | 64 | 1280 |
| LD69-1 | TLD69-1 | 8 | 4 | >64 | 4 | >64 | >64 | 8 | 2 | 64 | 64 | >128 | 4 | >128 | >128 | 1 | 0.008 | ≤0.004 | 0.008 | 64 | 1280 |
| LD70-1 | TLD70-1 | 4 | 4 | 4 | 4 | >64 | >64 | 1 | 2 | 64 | 64 | 16 | 0.5 | >128 | >128 | 0.03 | 0.008 | 0.008 | 0.03 | 64 | 1280 |
| LD70-2 | TLD70-2 | 4 | 4 | 4 | 4 | >64 | >64 | 1 | 2 | >64 | 64 | 16 | 1 | >128 | >128 | 0.015 | 0.004 | 0.015 | 0.015 | 64 | 1280 |
| LD72-1 | TLD72-1 | 4 | 4 | 8 | 4 | >64 | >64 | 4 | 2 | 64 | >64 | 4 | 2 | >128 | >128 | 0.015 | 0.008 | 0.008 | 0.015 | 128 | 1280 |
| LD73-1 | TLD73-1 | 4 | 4 | >64 | >64 | >64 | 32 | 1 | 2 | 0.5 | 2 | 64 | 8 | >128 | >128 | 0.03 | 0.008 | 0.008 | 0.008 | 128 | 1280 |
| LD75-1 | TLD75-1 | 4 | 4 | >64 | >64 | >64 | 32 | 16 | 2 | 1 | 2 | 4 | 16 | >128 | >128 | 8 | 0.008 | 0.008 | 0.008 | 64 | 1280 |
| LD75-2 | TLD75-2 | 4 | 4 | 8 | 4 | >64 | >64 | 2 | 2 | 64 | 64 | 8 | 2 | >128 | >128 | 0.03 | 0.004 | 0.008 | 0.015 | 128 | 1280 |
| LD76-1 | TLD76-1 | 4 | 4 | >64 | 4 | 4 | 1 | 64 | 2 | 1 | 0.25 | >128 | 2 | >128 | 8 | 16 | 0.008 | 0.015 | 0.015 | 128 | 1280 |
| LD78-1 | TLD78-1 | 4 | 4 | >64 | >64 | >64 | >64 | 1 | 2 | 1 | 4 | 32 | 4 | >128 | >128 | 0.015 | 0.008 | 0.008 | 0.015 | 64 | 1280 |
| LD81-1 | TLD81-1 | 8 | 4 | >64 | >64 | >64 | 16 | 1 | 2 | 2 | 2 | 64 | 2 | >128 | >128 | 0.03 | 0.008 | 0.008 | 0.008 | 256 | 1280 |
| LD84-1 | TLD84-1 | 4 | 4 | >64 | 4 | >64 | 0.5 | 1 | 2 | 1 | ≤0.125 | 16 | 4 | 128 | 16 | 0.06 | 0.008 | 0.008 | 0.015 | 64 | 1280 |
| LD85-1 | TLD85-1 | 4 | 4 | >64 | >64 | >64 | >64 | 4 | 2 | 1 | 2 | 64 | 8 | >128 | >128 | 0.008 | 0.015 | ≤0.004 | 0.015 | 64 | 1280 |
| LD86-1 | TLD86-1 | 4 | 4 | >64 | >64 | >64 | 32 | 16 | 2 | 1 | 4 | 2 | 16 | >128 | >128 | 4 | 0.015 | 0.008 | 0.015 | 128 | 1280 |
| LD87-1 | TLD87-1 | 8 | 4 | >64 | 4 | >64 | >64 | 16 | 2 | 64 | >64 | >128 | 2 | >128 | >128 | 1 | 0.008 | 0.008 | 0.008 | 64 | 1280 |
| LD91-1 | TLD91-1 | 4 | 4 | >64 | >64 | >64 | 64 | 16 | 16 | 2 | 4 | 32 | 4 | >128 | >128 | 1 | 0.008 | ≤0.004 | 0.015 | 64 | 1280 |
| LD91-2 | TLD91-2 | 4 | 4 | 4 | 4 | >64 | >64 | 4 | 2 | 64 | 32 | 16 | 4 | >128 | >128 | 0.03 | 0.008 | 0.015 | 0.008 | 64 | 1280 |
| LD92-2 | TLD92-2 | 4 | 4 | 4 | 8 | >64 | >64 | 1 | 2 | >64 | 64 | 16 | 0.5 | >128 | >128 | 0.008 | 0.008 | 0.008 | 0.015 | 128 | 1280 |
| LD93-1 | TLD93-1 | 4 | 4 | >64 | 8 | 1 | 1 | 8 | 2 | ≤0.125 | ≤0.125 | 32 | 0.5 | 128 | 4 | 0.06 | 0.008 | 0.008 | 0.015 | 128 | 1280 |
| LD94-1 | TLD94-1 | 4 | 4 | 4 | 8 | 1 | 0.5 | 2 | 2 | ≤0.125 | ≤0.125 | 4 | 4 | 4 | 4 | 0.03 | 0.008 | 0.008 | 0.008 | 128 | 1280 |
| LD94-2 | TLD94-2 | 4 | 4 | 4 | 4 | >64 | >64 | 1 | 2 | >64 | 64 | 16 | 4 | >128 | >128 | 0.015 | 0.008 | 0.008 | 0.008 | 128 | 1280 |
| LD95-1 | TLD95-1 | 4 | 4 | 4 | 4 | >64 | >64 | 1 | 1 | 32 | >64 | 8 | 0.5 | >128 | >128 | 0.008 | 0.008 | 0.008 | 0.015 | 64 | 1280 |
| LD96-1 | TLD96-1 | 4 | 4 | 4 | 8 | >64 | >64 | 2 | 2 | 32 | 64 | 4 | 4 | >128 | >128 | 0.015 | 0.004 | 0.015 | 0.008 | 128 | 1280 |
| LD97-2 | TLD97-2 | 8 | 4 | 4 | 8 | >64 | >64 | 1 | 2 | 32 | 64 | 16 | 2 | >128 | >128 | 0.03 | 0.008 | 0.008 | 0.03 | 64 | 1280 |
| ATCC25922 | | 0.25 | | 4 | | 1 | | 1 | | ≤0.125 | | 8 | | 4 | | 0.015 | | 0.008 | | 64 | |
| J53 | | 0.25 | | 4 | | 0.5 | | 2 | | ≤0.125 | | 0.5 | | 8 | | 0.008 | | 0.015 | | 1280 | |

**Abbreviations**: CST, colistin; FFC, florfenicol; CFF, ceftiofur; DOX, doxycycline; ATM, aztreonam; STR, streptomycin; AMC, amoxicillin; ENR, enrofloxacin; MEM, meropenem; NaN3, sodium azide.

P, Partental strains; T, Transconjugants.
