# Supplementary material for: Widespread Prevalence of Plasmid-Mediated Colistin Resistance Gene mcr-1 in Escherichia coli from Père David's Deer in China
Source: mSphere. 2020 Dec 23;5(6):e01221-20. doi: 10.1128/mSphere.01221-20 (PMC7763555; doi:10.1128/mSphere.01221-20)
Supplement: TABLE S2 [file mSphere.01221-20-st002.docx]

| IDs | | *mcr-1* gene | | Replicon sequence typing | | β-lactamases-producing genes | |
| --- | --- | --- | --- | --- | --- | --- | --- |
| Partental strains | Transconjugants | Partental strains | Transconjugants | Partental strains | Transconjugants | Partental strains | Transconjugants |
| LD3-1 | TLD3-1 | + | + | IncX4 | IncX4 | *bla*_OXA-1-like_ |  |
| LD4-1 | TLD4-1 | + | + | IncX4 | IncX4 | *bla*_OXA-1-like_ |  |
| LD4-2 | TLD4-2 | + | + | IncI2 | IncI2 | *bla*_CTX-M_ | *bla*_CTX-M_ |
| LD5-1 | TLD5-1 | + | + | IncI2/IncF/IncFIB | IncI2 | *bla*_CTX-M_ | *bla*_CTX-M_ |
| LD7-2 | TLD7-2 | + | + | IncHI2/IncN/IncFIB/IncF | IncHI2/IncN | *bla*_CTX-M_, *bla*_TEM_ | *bla*_CTX-M_ |
| LD8-1 | TLD8-1 | + | + | IncI2 | IncI2 | *bla*_CTX-M_ | *bla*_CTX-M_ |
| LD9-1 | TLD9-1 | + | + | IncX4 | IncX4 | *bla*_OXA-1-like_ |  |
| LD9-2 | TLD9-2 | + | + | IncI2 | IncI2 | *bla*_CTX-M_ | *bla*_CTX-M_ |
| LD13-1 | TLD13-1 | + | + | IncX4 | IncX4 | *bla*_OXA-1-like_ |  |
| LD14-1 | TLD14-1 | + | + | IncI2/IncHI1 | IncI2 |  |  |
| LD16-1 | TLD16-1 | + | + | IncX4 | IncX4 | *bla*_OXA-1-like_ |  |
| LD18-1 | TLD18-1 | + | + | IncI2/IncF/IncFIB | IncI2 | *bla*_CTX-M_ | *bla*_CTX-M_ |
| LD20-1 | TLD20-1 | + | + | IncI2 | IncI2 | *bla*_CTX-M_ | *bla*_CTX-M_ |
| LD22-1 | TLD22-1 | + | + | IncHI2/IncN/IncFIB/IncF | IncHI2/IncN | *bla*_CTX-M_, *bla*_TEM_ | *bla*_CTX-M_ |
| LD24-1 | TLD24-1 | + | + | IncHI2/IncF/IncFIB/IncI2 | IncI2 | *bla*_CTX-M_, *bla*_TEM_ | *bla*_CTX-M_ |
| LD24-2 | TLD24-2 | + | + | IncI2 | IncI2 | *bla*_CTX-M_ |  |
| LD25-1 | TLD25-1 | + | + | IncI2/IncF/IncFIB | IncI2 | *bla*_CTX-M_ | *bla*_CTX-M_ |
| LD26-1 | TLD26-1 | + | + | IncHI2/IncN/IncFIB/IncF | IncHI2/IncN | *bla*_CTX-M_, *bla*_TEM_ | *bla*_CTX-M_ |
| LD26-2 | TLD26-2 | + | + | IncI2 | IncI2 | *bla*_CTX-M_ | *bla*_CTX-M_ |
| LD27-1 | N | + | N | N | N | *bla*_TEM_ |  |
| LD28-1 | TLD28-1 | + | + | IncHI2/IncN/IncI2 | IncHI2/IncN | *bla*_CTX-M_ | *bla*_CTX-M_ |
| LD31-2 | TLD31-2 | + | + | IncI2 | IncI2 | *bla*_CTX-M_ | *bla*_CTX-M_ |
| LD36-1 | TLD36-1 | + | + | IncHI2/IncF/IncFIB/IncI2 | IncI2 | *bla*_CTX-M_, *bla*_TEM_ | *bla*_CTX-M_ |
| LD36-2 | TLD36-2 | + | + | IncI2 | IncI2 | *bla*_CTX-M_ | *bla*_CTX-M_ |
| LD37-1 | TLD37-1 | + | + | IncI2/IncF/IncFIB | IncI2 | *bla*_CTX-M_ |  |
| LD38-1 | TLD38-1 | + | + | IncHI1/IncI2/IncFIB | IncI2 | *bla*_CTX-M_, *bla*_TEM_ | *bla*_CTX-M_ |
| LD38-2 | TLD38-2 | + | + | IncI2 | IncI2 | *bla*_CTX-M_ | *bla*_CTX-M_ |
| LD39-1 | TLD39-1 | + | + | IncHI2/IncN /IncFIA/FIB | IncHI2/IncN | *bla*_CTX-M_ | *bla*_CTX-M_ |
| LD39-2 | TLD39-2 | + | + | IncI2 | IncI2 | *bla*_CTX-M_ | *bla*_CTX-M_ |
| LD40-1 | TLD40-1 | + | + | IncHI2/IncF/IncFIB/IncI2 | IncI2 | *bla*_CTX-M_, *bla*_TEM_ | *bla*_CTX-M_ |
| LD41-1 | TLD41-1 | + | + | IncHI2/IncF/IncFIB/IncI2 | IncI2 | *bla*_CTX-M_, *bla*_TEM_ | *bla*_CTX-M_ |
| LD42-1 | TLD42-1 | + | + | IncHI2/IncF/IncFIB/IncI2 | IncI2 | *bla*_CTX-M_, *bla*_TEM_ | *bla*_CTX-M_ |
| LD47-2 | TLD47-2 | + | + | IncI2 | IncI2 | *bla*_CTX-M_ | *bla*_CTX-M_ |
| LD48-1 | TLD48-1 | + | + | IncI1/IncF/IncFIB/IncI2 | IncI2 | *bla*_CTX-M_ |  |
| LD50-1 | TLD50-1 | + | + | IncHI2/IncN | IncHI2/IncN | *bla*_CTX-M_ | *bla*_CTX-M_ |
| LD51-1 | TLD51-1 | + | + | IncI2/IncF/IncFIB | IncI2 | *bla*_CTX-M_ | *bla*_CTX-M_ |
| LD52-1 | TLD52-1 | + | + | IncI2 | IncI2 | *bla*_CTX-M_ |  |
| LD53-2 | TLD53-2 | + | + | IncI2 | IncI2 | *bla*_CTX-M_ | *bla*_CTX-M_ |
| LD54-1 | TLD54-1 | + | + | IncHI2/IncN | IncHI2/IncN | *bla*_CTX-M_ | *bla*_CTX-M_ |
| LD54-2 | TLD54-2 | + | + | IncI2 | IncI2 | *bla*_CTX-M_ | *bla*_CTX-M_ |
| LD55-2 | TLD55-2 | + | + | IncI2 | IncI2 | *bla*_CTX-M_ |  |
| LD65-1 | TLD65-1 | + | + | IncX4 | IncX4 |  |  |
| LD67-1 | TLD67-1 | + | + | IncHI2/IncF/IncFIB/IncI2 | IncI2 | *bla*_CTX-M_, *bla*_TEM_ | *bla*_CTX-M_ |
| LD68-2 | TLD68-2 | + | + | IncI2 | IncI2 | *bla*_CTX-M-1G_ | *bla*_CTX-M_ |
| LD69-1 | TLD69-1 | + | + | IncHI2/IncF/IncFIB/IncI2 | IncI2 | *bla*_CTX-M_, *bla*_TEM_ | *bla*_CTX-M_ |
| LD70-1 | TLD70-1 | + | + | IncI2 | IncI2 | *bla*_CTX-M_ | *bla*_CTX-M_ |
| LD70-2 | TLD70-2 | + | + | IncI2 | IncI2 | *bla*_CTX-M_ | *bla*_CTX-M_ |
| LD72-1 | TLD72-1 | + | + | IncI2/IncF/IncFIB | IncI2 | *bla*_CTX-M_ | *bla*_CTX-M_ |
| LD73-1 | TLD73-1 | + | + | IncHI2/IncN | IncHI2/IncN | *bla*_CTX-M_ | *bla*_CTX-M_ |
| LD75-1 | TLD75-1 | + | + | IncHI2/IncN/IncFIB/IncF | IncHI2/IncN | *bla*_CTX-M_, *bla*_TEM_ | *bla*_CTX-M_ |
| LD75-2 | TLD75-2 | + | + | IncI2 | IncI2 | *bla*_CTX-M_ | *bla*_CTX-M_ |
| LD76-1 | TLD76-1 | + | + | IncHI2/IncF/IncFIB/IncI2 | IncI2 | *bla*_CTX-M_, *bla*_TEM_ |  |
| LD78-1 | TLD78-1 | + | + | IncHI2/IncN/IncFIB/IncF | IncHI2/IncN | *bla*_CTX-M_, *bla*_TEM_ | *bla*_CTX-M_ |
| LD81-1 | TLD81-1 | + | + | IncHI2/IncN/IncFIB/IncF | IncHI2/IncN | *bla*_CTX-M_, *bla*_TEM_ | *bla*_CTX-M_ |
| LD84-1 | TLD84-1 | + | + | IncX4, | IncX4 | *bla*_OXA-1-like_ |  |
| LD85-1 | TLD85-1 | + | + | IncHI2/IncN | IncHI2/IncN | *bla*_CTX-M_ | *bla*_CTX-M_ |
| LD86-1 | TLD86-1 | + | + | IncHI2/IncN/IncFIB/IncF | IncHI2/IncN | *bla*_CTX-M_, *bla*_TEM_ | *bla*_CTX-M_ |
| LD87-1 | TLD87-1 | + | + | IncHI2/IncF/IncFIB/IncI2 | IncI2 | *bla*_CTX-M_, *bla*_TEM_ | *bla*_CTX-M_ |
| LD91-1 | TLD91-1 | + | + | IncHI2/IncN/IncFIB/IncF | IncHI2/IncN/IncFIB/IncF | *bla*_CTX-M_, *bla*_TEM_ | *bla*_CTX-M_, *bla*_TEM_ |
| LD91-2 | TLD91-2 | + | + | IncI2/IncFIB | IncI2 | *bla*_CTX-M_ | *bla*_CTX-M_ |
| LD92-2 | TLD92-2 | + | + | IncI2 | IncI2 | *bla*_CTX-M_ | *bla*_CTX-M_ |
| LD93-1 | TLD93-1 | + | + | IncX4 | IncX4 | *bla*_OXA-1-like_ |  |
| LD94-1 | TLD94-1 | + | + | IncI2 | IncI2 | *bla*_CTX-M_ |  |
| LD94-2 | TLD94-2 | + | + | IncI2 | IncI2 | *bla*_CTX-M_ | *bla*_CTX-M_ |
| LD95-1 | TLD95-1 | + | + | IncI2 | IncI2 | *bla*_CTX-M_ | *bla*_CTX-M_ |
| LD96-1 | TLD96-1 | + | + | IncI2 | IncI2 | *bla*_CTX-M_ | *bla*_CTX-M_ |
| LD97-2 | TLD97-2 | + | + | IncI2 | IncI2 | *bla*_CTX-M_ | *bla*_CTX-M_ |

Note: “+” : *mcr-1* gene positive. N: No transconjugants was acquired and no *mcr-1* gene or replicons were identified. *mcr-1* was located in the chromosome in strain LD27-1.
