# Supplementary material for: Widespread Prevalence of Plasmid-Mediated Colistin Resistance Gene mcr-1 in Escherichia coli from Père David's Deer in China
Source: mSphere. 2020 Dec 23;5(6):e01221-20. doi: 10.1128/mSphere.01221-20 (PMC7763555; doi:10.1128/mSphere.01221-20)
Supplement: TABLE S3 [file mSphere.01221-20-st003.docx]

| β-lactamases-producing genes | Number of positive strains | Positive rate |
| --- | --- | --- |
| *bla*_CTX-M_ | 57 | 85.07% |
| *bla*_TEM_ | 19 | 28.35% |
| Coexistence of *bla*_CTX-M_ and *bla*_TEM_ | 18 | 26.87% |
| *bla*_OXA-1-like_ | 7 | 10.45% |
| Total | 65 | 97.01% |
