# Supplementary figures and images for: Widespread Prevalence of Plasmid-Mediated Colistin Resistance Gene mcr-1 in Escherichia coli from Père David's Deer in China
Source: mSphere. 2020 Dec 23;5(6):e01221-20. doi: 10.1128/mSphere.01221-20 (PMC7763555; doi:10.1128/mSphere.01221-20)

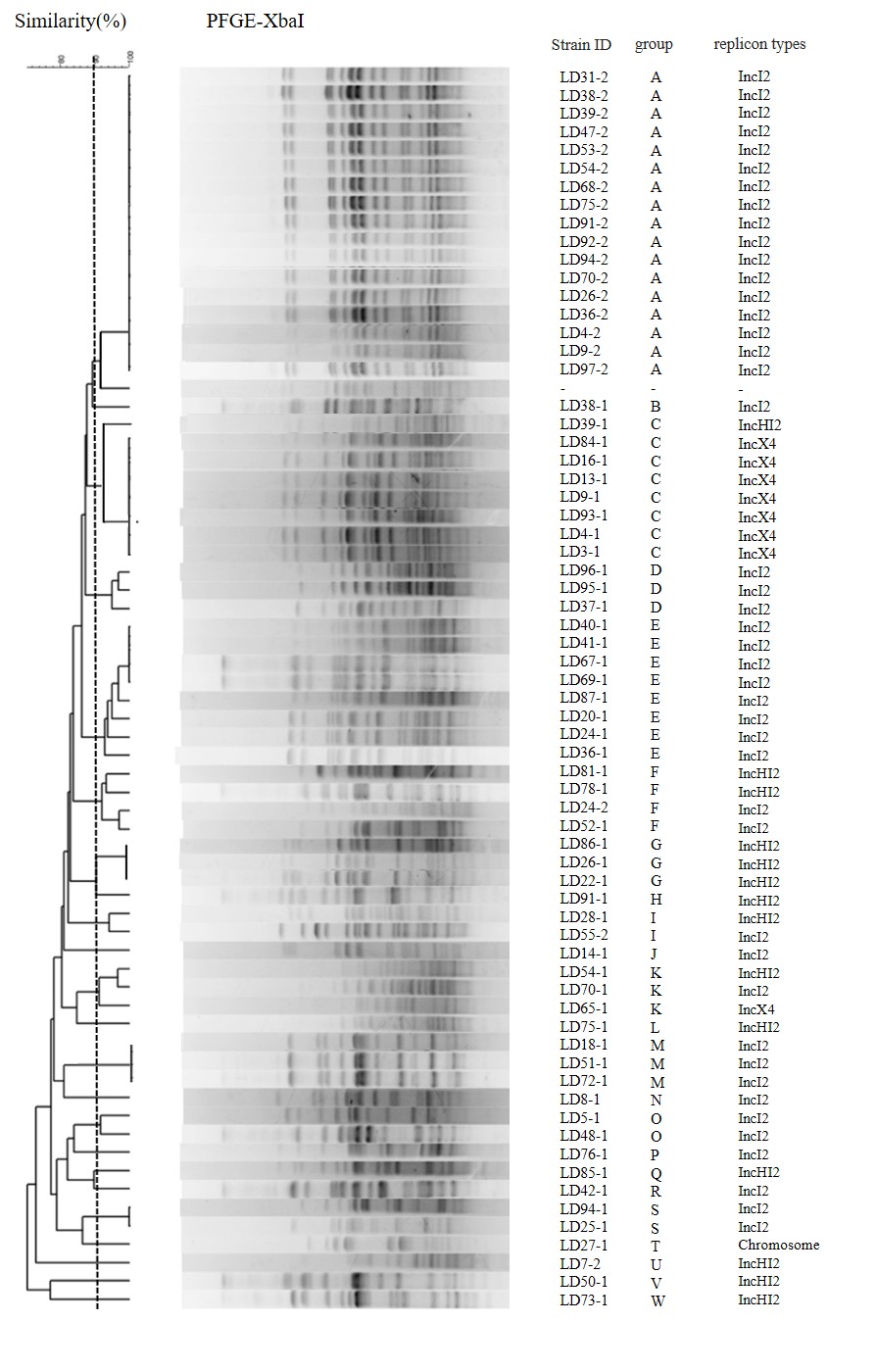

Supplement: FIG S1 [file mSphere.01221-20-sf001.jpg]

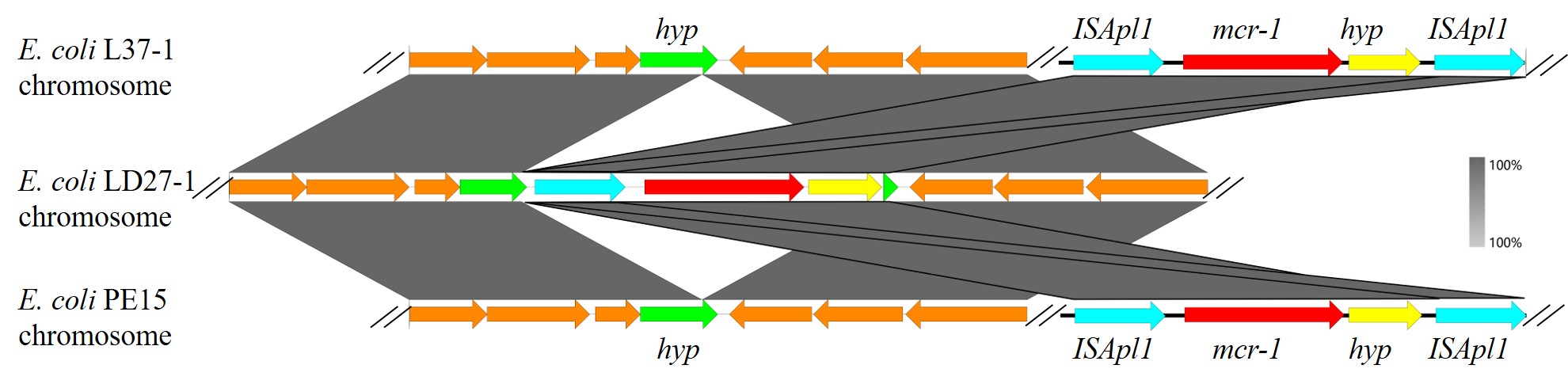

Supplement: FIG S2 [file mSphere.01221-20-sf002.jpg]
